# Systematic analysis of gene expression patterns associated with postmortem interval in human tissues

**DOI:** 10.1038/s41598-017-05882-0

**Published:** 2017-07-14

**Authors:** Yizhang Zhu, Likun Wang, Yuxin Yin, Ence Yang

**Affiliations:** 1 Institute of Systems Biomedicine, School of Basic Medical Sciences, Peking University Health Science Center, Beijing, 100191 China; 2 Department of Pathology, School of Basic Medical Sciences, Peking University Health Science Center, Beijing, 100191 China; 3 Department of Microbiology, School of Basic Medical Sciences, Peking University Health Science Center, Beijing, 100191 China

## Abstract

Postmortem mRNA degradation is considered to be the major concern in gene expression research utilizing human postmortem tissues. A key factor in this process is the postmortem interval (PMI), which is defined as the interval between death and sample collection. However, global patterns of postmortem mRNA degradation at individual gene levels across diverse human tissues remain largely unknown. In this study, we performed a systematic analysis of alteration of gene expression associated with PMI in human tissues. From the Genotype-Tissue Expression (GTEx) database, we evaluated gene expression levels of 2,016 high-quality postmortem samples from 316 donors of European descent, with PMI ranging from 1 to 27 hours. We found that PMI-related mRNA degradation is tissue-specific, gene-specific, and even genotype-dependent, thus drawing a more comprehensive picture of PMI-associated gene expression across diverse human tissues. Additionally, we also identified 266 differentially variable (DV) genes, such as DEFB4B and IFNG, whose expression is significantly dispersed between short PMI (S-PMI) and long PMI (L-PMI) groups. In summary, our analyses provide a comprehensive profile of PMI-associated gene expression, which will help interpret gene expression patterns in the evaluation of postmortem tissues.

## Introduction

Human postmortem tissue, representing a type of valuable biological material, is widely used in various fields of study including biology, pathology, and forensic medicine^1–3^. Research using postmortem tissues from autopsies has been fundamental for improving knowledge of many diseases, including neurological disorders in particular^4, 5^. However, use of such tissue invariably involves a time delay, as tissue samples cannot immediately be stored in conditions which prevent mRNA degradation. As a result, the probability of cell autolysis and RNA degradation increases, potentially compromising the gene expression data. Thus, the major concern regarding the utility of autopsy tissue is postmortem mRNA degradation and how accurately postmortem mRNA represents physiologic conditions.

Postmortem mRNA degradation is a complex process affected by many factors^6, 7^, including agonal state, pH, and postmortem interval. The postmortem interval (PMI) between death and sample collection is an important factor. Previous studies have reported that RNA degradation is associated with PMI in many species^8–10^, including human tissues^11–13^. Nevertheless, these studies had limitations. First, some of these samples were of poor quality with low RNA integrity number (RIN) scores. RNA integrity number (RIN) is an electrophoretic-basic method for evaluating the 28S to 18S ribosomal RNA (rRNA) ratio^14^, which reflects RNA integrity as a whole. Potential bias may result if RNA of low RINs is not well extracted. Second, these studies focused mainly on the correlation of RIN and PMI. However, RIN is largely derived based on the integrity of rRNA, and mRNA degradation may be distinctly different and gene specific, which is not completely reflected by the RIN. Third, previous research focused on the expression of several genes associated with PMI using limited methods, such as quantitative reverse transcription-PCR (qRT-PCR). Only one of these studies employed high-throughput sequencing technologies^15^. In addition, the sample size in these studies was small, and ranged from several to a dozen specimens, due to the difficulty in sample collection of human tissue.

The Genotype-Tissue Expression (GTEx) project^16^ provides an opportunity to systemically investigate the genome-wide patterns of change in gene expression levels associated with PMI in various human postmortem tissues. This project was initiated by the National Institutes of Health (NIH) to determine how genetic variation affects normal gene expression in human tissues, and thus ultimately inform the study of human disease. A large number of postmortem tissues were collected, including more than 50 different types of tissue from hundreds of human donors unselected for any disease. High-quality postmortem tissues were used to isolate nucleic acids, and genotyping, gene expression profiling, whole genome sequencing, and RNA sequencing (RNA-seq) analysis were performed. Accurate postmortem intervals were recorded for a majority of samples, ranging from 1 to 27 hours. The GTEx database is therefore well suited for transcriptome research into how PMI affects the gene expression across diverse postmortem human tissues.

In this study, we present a comprehensive view of mRNA degradation associated with PMI in 15 human tissues using the GTEx RNA-seq data set. We found that postmortem mRNA degradation occurs in a tissue-type specific manner and is associated with gene-specific properties. mRNAs are relatively stable in central nervous system (CNS) tissues but are unstable in postmortem digestive tract tissues. Functional annotation analysis indicates that the mechanism for mRNA degradation in postmortem tissues is neither a wholly normal cellular function nor a random process. We also evaluated the interaction between genotype and PMI to access the influence of genetic background on postmortem mRNA degradation in whole blood, which showed that postmortem degradation occurs in a genotype-specific manner. Additionally, we identified 266 differentially variable (DV) genes associated with prolonged PMI by using Levene’s test. These findings provide a picture of gene expression affected by PMI in different human postmortem tissues, which may serve to improve the use of postmortem tissue for research.

## Results

### Identification of tissue-specific PMI-associated genes in the GTEx data

**Figure 1:**
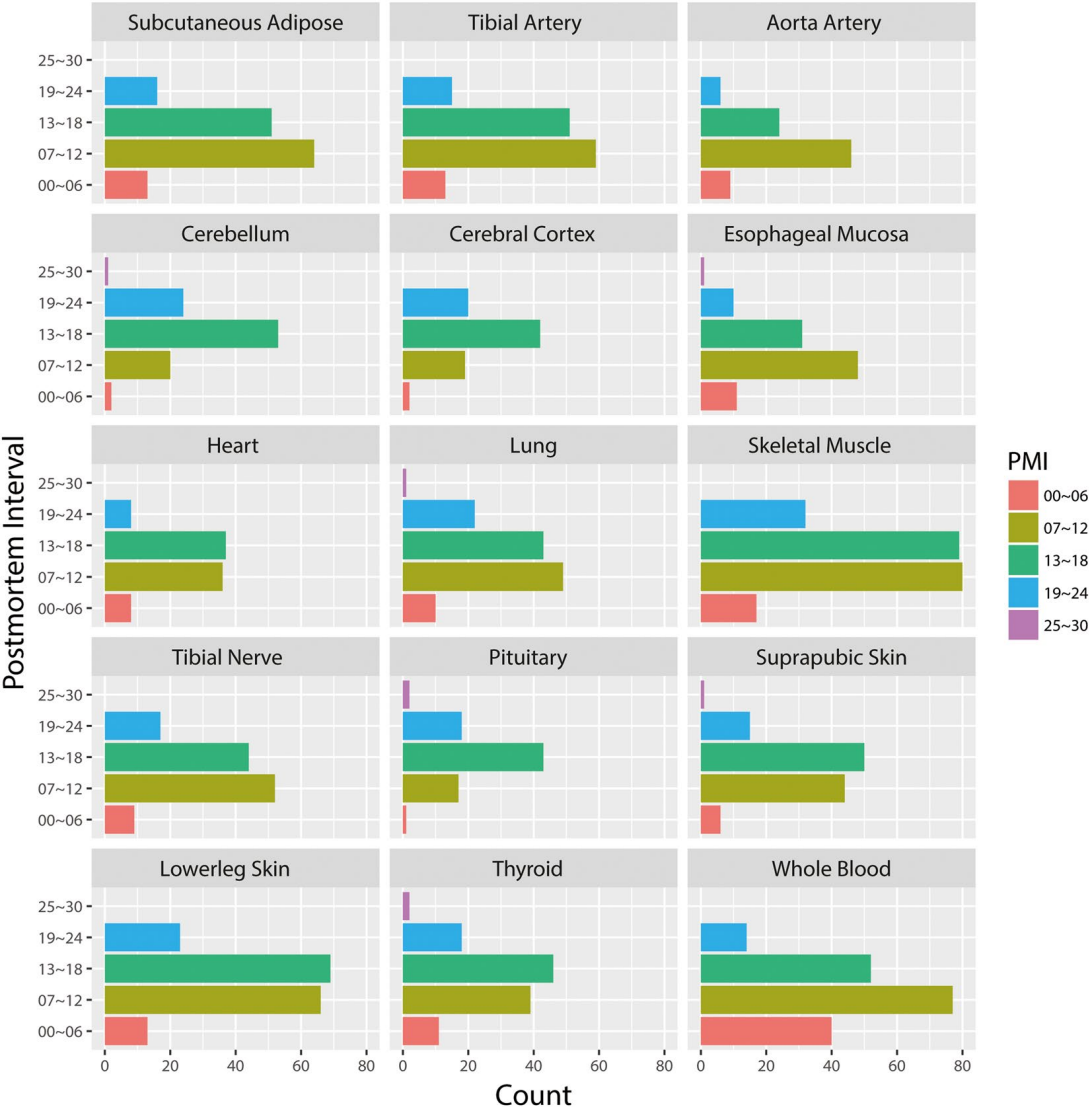
Postmortem Interval distribution of samples in 15 human tissues. Each histogram denotes the distribution of samples with different PMIs in hours.

The Genotype-Tissue Expression (GTEx) project is a resource project to discover expression quantitative trait loci (eQTL) using data from 900 donors in more than 53 sampled sites^16^. At an interim point in this project, the transcriptomic profiles from 573 donors were obtained from the GTEx portal website. We next excluded samples derived from non-postmortem sources and non-European donors. It is of note that very few samples were available for some types of tissue (e.g., vagina, spleen, and bladder), which may reduce confidence in some conclusions. We thus filtered out tissues with less than 80 samples. We then obtained a total of 2,016 samples from 15 distinct tissue types (or subtypes), including (1) adipose (subcutaneous), (2) aorta artery, (3) tibial artery, (4) cerebellum, (5) cerebral cortex, (6) esophageal mucosa, (7) heart (atrial appendage), (8) lung, (9) skeletal muscle, (10) tibial nerve, (11) pituitary, (12) skin (suprapubic), (13) skin (lower leg), (14) thyroid, and (15) whole blood. The distribution of the postmortem interval was plotted for 15 human tissues (Fig. 1), showing that the PMI ranged from 1 to 27 hours. The final data matrices, including 81 to 208 samples all from donors of European descent, were separately normalized in different tissues (see Methods).

To identify PMI-associated genes, we used multiple linear regression models, controlling covariates and hidden confounding factors (see Methods). To reduce false positives, we performed the permutation test 10,000 times (see Methods). We summarized the numbers of PMI-associated genes in 15 human tissues with up-regulated and down-regulated at the false discovery rate (FDR) of 1, 5, and 10%, respectively (Table 1). At an FDR of 5%, 7,546 distinct genes were identified as PMI-associated genes from at least one tissue, while the number of PMI-associated genes for each tissue varied dramatically from 0 to 2,763. The details of PMI-associated genes from each tissue are provided in Supplementary Data S1. Among these, 1, 14, 96, 482, and 1,922 genes were found in six, five, four, three, and two tissues respectively. For each of these “multi-hit” genes, the direction of the expression response to the PMI was the same in the different tissues where the gene was identified. These genes appear to be involved in some fundamental metabolic processes or pathways associated with PMI, and this finding can be applied to multiple tissues. For example, CAPN5, the calcium-dependent cysteine protease which is ubiquitously expressed in mammals^17^, was found to be upregulated in five tissues.

**Table 1:** Numbers of PMI-associated genes in 15 human tissues.

Tissues	FDR < 1%			FDR < 5%			FDR < 10%		
	up	down	total	up	down	total	up	down	total
(1) Cerebellum (*n* = 100)	0	0	**0**	0	0	**0**	0	0	**0**
(2) Pituitary (*n* = 81)	0	0	**0**	0	0	**0**	0	0	**0**
(3) Subcutaneous Adipose (*n* = 144)	3	3	**6**	6	5	**11**	8	5	**13**
(4) Suprapubic Skin (*n* = 116)	10	14	**24**	19	26	**45**	29	32	**61**
(5) Cerebral Cortex (*n* = 83)	2	8	**10**	41	64	**105**	124	138	**262**
(6) Lung (*n* = 125)	40	78	**118**	56	100	**156**	68	113	**181**
(7) Tibial Artery (*n* = 138)	49	67	**116**	85	109	**194**	113	144	**257**
(8) Tibial Nerve (*n* = 122)	165	70	**235**	228	110	**338**	252	129	**381**
(9) Lower leg Skin (*n* = 171)	198	159	**357**	351	285	**636**	485	366	**851**
(10) Thyroid (*n* = 116)	225	223	**448**	317	333	**650**	378	390	**768**
(11) Heart (*n* = 89)	199	200	**399**	421	405	**826**	582	575	**1157**
(12) Aorta Artery(*n* = 85)	430	494	**924**	693	720	**1413**	853	844	**1697**
(13) Skeletal Muscle (*n* = 208)	653	600	**1253**	843	811	**1654**	963	922	**1885**
(14) Whole Blood (*n* = 183)	677	842	**1519**	884	1106	**1990**	1021	1237	**2258**
(15) Esophageal Mucosa (*n* = 101)	1129	1020	**2149**	1529	1234	**2763**	1732	1355	**3087**

Columns “up” and “down” list the number of positive correlation and negative correlation PMI-associated genes respectively. Results derived from using three different FDR cutoffs (1%, 5%, and 10%) are shown.

**Figure 2:**
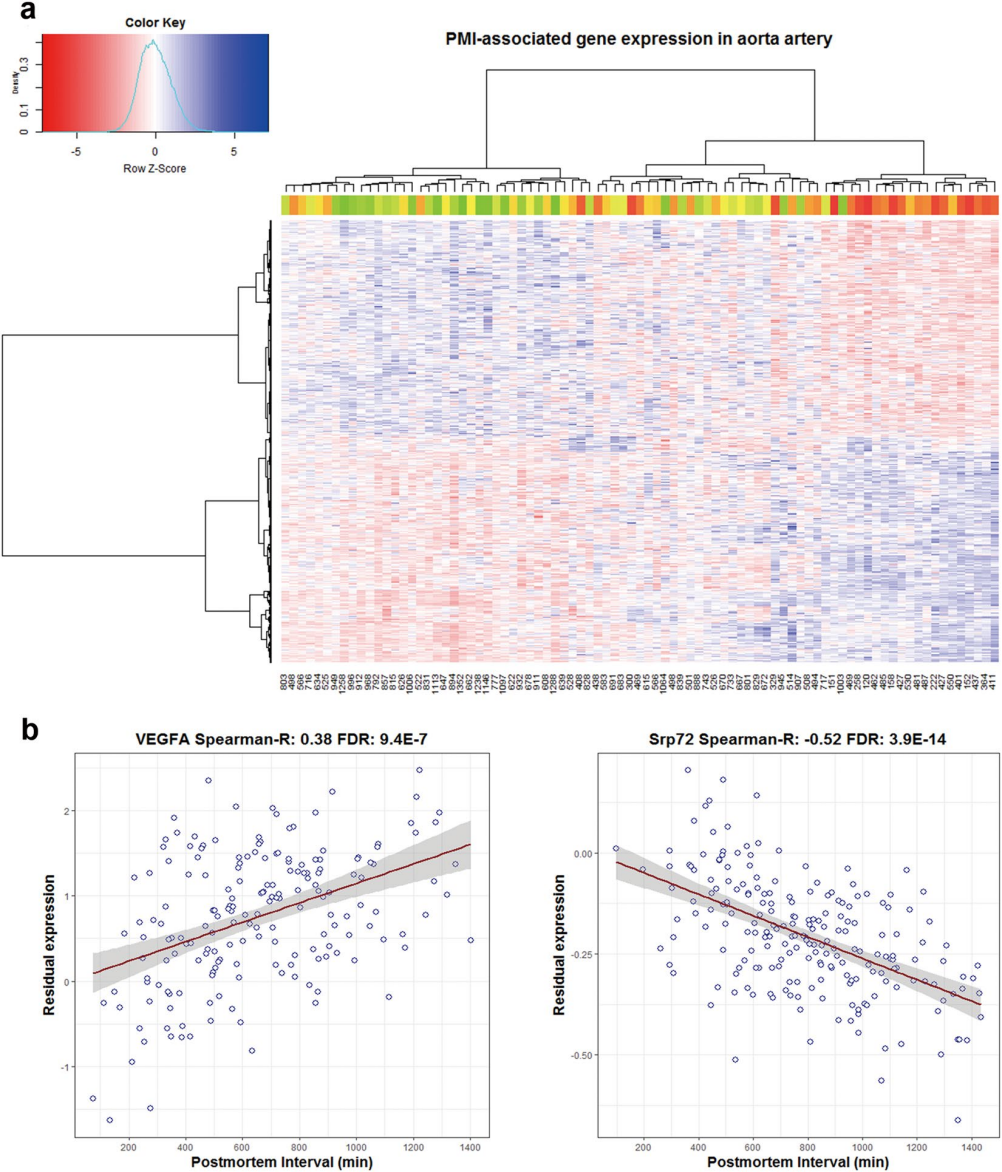
Visualization of gene expression associated with PMI. (**a**) Heat map of 1,413 PMI-associated genes (row) on 85 samples (column). Colors represent residual gene expression values with red for low expression and blue for high expression. The PMI of each individual is displayed at the bottom and also illustrated in color bar at the top with color changes from red to green with PMI increased. (**b**) Scatter plot of two representative PMI-associated gene expression patterns of VEGFA and Srp72 in whole blood and muscle skeletal respectively. The dots represent the expression of samples. Spearman-R value in the title represents the Spearman correlation coefficient of gene expression and PMI across all samples.

In four tissues (cerebellum, pituitary, subcutaneous adipose, and suprapubic skin) only a few PMI-associated genes were identified, and we plotted the heat map of expression of these PMI-associated genes across samples in the remaining 11 tissues, whose number of identified PMI-associated genes was larger than 100 (Fig. S1). As shown in Fig. 2a, aortic samples were clearly clustered into Short-PMI (S-PMI) and Long-PMI (L-PMI) groups using the Euclidian distance with “Ward” measurement^18^. The mean time of PMI in the right-side group was much shorter than that in the left-side group. This apparent separation of “S-PMI” and “L-PMI” groups was observed in all 11 tissues. Furthermore, the Student’s *t*-test was performed to compare these groups and gave significant results in every instance (*P*-values < 5.8E-3). Details are provided in Supplementary Table S1. To validate gene expression associated with PMI at individual gene levels, we selected two representative genes with either significantly positive or negative correlation (VEGFA and Srp72) in whole blood and skeletal muscle respectively (Fig. 2b). In association with increasing PMI, the residual expression of VEGFA in whole blood showed an upward trend (Spearman-R = 0.38; FDR = 9.40E-7) while Srp72 showed the reverse pattern in muscle skeletal (Spearman-R = −0.52; FDR = 3.86E-14). These trends are consistent with previous studies verified by quantitative reverse transcription-PCR (qRT-PCR)^12, 19^.

In view of the fact that sample size may have a marked impact on the numbers of PMI-associated genes identified, we cannot directly compare the numbers of identified PMI-associated genes in tissues of different sample sizes. After correction for sample size effect (see Methods), we observed that some tissues still have more significantly PMI-associated genes than other tissue (Table S2), suggesting different tissues may have different levels of sensitivity or susceptibility to postmortem mRNA degradation. The CNS (cerebellum, cerebral cortex, and pituitary), as well as the lung, showed no (or less) sensitivity to postmortem mRNA degradation, which is consistent with previous studies^6, 20, 21^. However, the esophageal mucosa showed the greatest sensitivity to postmortem mRNA degradation, where 2,763 genes associated with PMI were identified in 101 individuals. It is of note that the esophageal mucosa belongs to the digestive tract, and the increased rate of tissue turnover or the presence of digestive enzymes in the digestive tract may contribute to its overall RNA degradation^11^.

### Functional annotations of PMI-associated genes

To acquire a functional overview of the biological processes or pathways involved in the PMI-related mRNA degradation across various human tissues, we systematically investigated the functional annotations of the up- and down-regulated PMI-associated genes separately in 15 human tissues. As described below, 162 highly significant GO terms of Biological Process and Molecular Function or the KEGG pathways were identified in eight tissues (FDR < 0.05, fold enrichment > 2). The complete list is provided in Supplementary Data S2. We found that *nuclear-transcribed mRNA catabolic process: nonsense-mediated decay* (GO: 0000184), one of the known mRNA degradation pathways was enriched in the top up-regulated significant annotations of tibial artery, esophageal mucosa, and thyroid (Table 2), suggesting that postmortem mRNA degradation in these tissues may be carried out in this manner.

**Table 2:** Function enrichment of up- and down-regulated PMI-associated genes in eight human tissues.

Tissues	Up-regulated gene set			Down-regulated gene set		
	ID	Description	FDR	ID	Description	FDR
**Aorta Artery**	GO:0042752	regulation of circadian rhythm	4.44E-02	GO:0006954	inflammatory response	3.31E-09
				GO:0002250	adaptive immune response	1.29E-08
				GO:0045087	innate immune response	2.27E-08
				GO:0002224	toll-like receptor signaling pathway	6.25E-04
				hsa04380	Osteoclast differentiation	2.20E-09
				hsa05150	Staphylococcus aureus infection	7.81E-09
**Tibial Artery**	GO:0006413	translational initiation	6.00E-07			
	GO:0006614	SRP-dependent cotranslational protein targeting to membrane	1.10E-05			
	GO:0006412	translation	1.84E-05			
	GO:0019083	viral transcription	4.41E-05			
	GO:0000184	nuclear-transcribed mRNA catabolic process, nonsense-mediated decay	7.11E-05			
	GO:0003735	structural constituent of ribosome	8.86E-05			
	hsa03010	Ribosome	1.21E-04			
**Esophageal Mucosa**	GO:0000184	nuclear-transcribed mRNA catabolic process, nonsense-mediated decay	2.43E-09	GO:0002223	stimulatory C-type lectin receptor signaling pathway	1.16E-06
	GO:0006614	SRP-dependent cotranslational protein targeting to membrane	9.62E-09	GO:0002479	antigen processing and presentation of exogenous peptide antigen via MHC class I, TAP-dependent	3.56E-06
	GO:0019083	viral transcription	5.20E-08	GO:0006511	ubiquitin-dependent protein catabolic process	4.75E-06
	GO:0006412	translation	1.72E-06	GO:0038061	NIK/NF-kappaB signaling	8.87E-06
	GO:0006413	translational initiation	5.15E-05	hsa04141	Protein processing in endoplasmic reticulum	7.36E-07
	GO:0003735	structural constituent of ribosome	1.03E-06	hsa04145	Phagosome	7.43E-06
	hsa03010	Ribosome	2.38E-09	hsa03050	Proteasome	8.65E-04
**Heart**	hsa00190	Oxidative phosphorylation	7.93E-03	GO:0006457	protein folding	8.57E-04
	hsa05012	Parkinson’s disease	1.95E-02	GO:0006954	inflammatory response	1.95E-03
	hsa05010	Alzheimer’s disease	4.02E-05	GO:1904871	positive regulation of protein localization to Cajal body	2.56E-02
	hsa05016	Huntington’s disease	1.93E-03	hsa05150	Staphylococcus aureus infection	9.61E-09
				hsa04380	Osteoclast differentiation	1.80E-05
				hsa04610	Complement and coagulation cascades	2.93E-03
**Skeletal Muscle**	GO:0006120	mitochondrial electron transport, NADH to ubiquinone	1.49E-25	GO:0043488	regulation of mRNA stability	1.87E-07
	GO:0032981	mitochondrial respiratory chain complex I assembly	1.74E-22	GO:0038061	NIK/NF-kappaB signaling	1.68E-03
	GO:0070125	mitochondrial translational elongation	8.12E-19	GO:0016032	viral process	2.56E-03
	GO:0070126	mitochondrial translational termination	2.05E-16	GO:0036498	IRE1-mediated unfolded protein response	2.59E-03
	hsa05012	Parkinson’s disease	2.88E-44	GO:0002223	stimulatory C-type lectin receptor signaling pathway	6.43E-03
	hsa00190	Oxidative phosphorylation	1.18E-42	GO:0044822	poly(A) RNA binding	2.57E-16
	hsa05016	Huntington’s disease	2.07E-34	hsa03050	Proteasome	7.14E-04
**Tibial Nerve**	GO:0007155	cell adhesion	3.42E-06	GO:0044822	poly(A) RNA binding	1.86E-02
	GO:0007399	nervous system development	8.04E-04			
	GO:0030198	extracellular matrix organization	1.43E-03			
	GO:0030199	collagen fibril organization	1.17E-02			
	hsa04514	Cell adhesion molecules (CAMs)	3.30E-03			
**Thyroid**	GO:0000184	nuclear-transcribed mRNA catabolic process, nonsense-mediated decay	7.33E-10			
	GO:0006413	translational initiation	8.10E-10			
	GO:0006614	SRP-dependent cotranslational protein targeting to membrane	3.80E-07			
	GO:0019083	viral transcription	4.07E-06			
	GO:0006412	translation	3.37E-05			
	GO:0003735	structural constituent of ribosome	7.78E-04			
	hsa03010	Ribosome	2.97E-06			
**Whole Blood**	GO:0003714	transcription corepressor activity	4.41E-02	GO:0051301	cell division	3.85E-08
	hsa04660	T cell receptor signaling pathway	5.11E-03	GO:0007067	mitotic nuclear division	1.01E-05
				GO:0043161	proteasome-mediated ubiquitin-dependent protein catabolic process	1.57E-05
				GO:0051436	negative regulation of ubiquitin-protein ligase activity involved in mitotic cell cycle	3.02E-05
				GO:0000082	G1/S transition of mitotic cell cycle	3.43E-05
				GO:0000083	regulation of transcription involved in G1/S transition of mitotic cell cycle	4.91E-04
				hsa04110	Cell cycle	2.64E-05

In the up-regulated PMI-associated genes overall, the most enriched GO terms or KEGG pathways included: *translation* (GO:0006412), *translational initiation* (GO:0006413), *structural constituent of ribosome* (GO:0003735), and *Ribosome* (hsa03010), which were identified in three tissues, indicating that mRNAs involved in protein synthesis and ribosome function are more stable and less sensitive to PMI-related mRNA degradation. This result is consistent with the findings of Yang *et. al*.^22^, which showed that mRNAs for biosynthetic proteins have lower average decay rates, and tend to be more stable during the PMI. In the down-regulated PMI-associated genes, the most related GO terms or KEGG pathways included: *inflammatory response* (GO:0006954), *NIK/NF-kappaB signaling* (GO:0038061), and *Proteasome* (hsa03050), indicating that mRNAs involved in the immune response and proteolysis are more susceptible to PMI-related mRNA degradation. Gupta *et al*.^23^ performed GO Slim terms annotation in postmortem cardiac tissues and identified the GO terms of *immune response* (GO:0006955), *defense response* (GO:0006952), and *inflammatory response* (GO:0006954) in the down-regulated gene list. We observed similar results in our annotation of heart (atrial appendage).

In addition, we found some unique GO terms in specific tissues which had not been previously reported. For example, we observed significant GO terms of *cell adhesion* (GO:0007155), *extracellular matrix organization* (GO:0030198), and *collagen fibril organization* (GO:0030199) in up-regulated genes of tibial nerve, indicating that mRNAs involved in these specific GO terms of cell adhesion are more stable and less sensitive to PMI-related mRNA degradation in tibial nerve. Many cell cycles related GO terms were annotated in down-regulated PMI-associated genes from whole blood, such as *cell division* (GO:0051301), *mitotic nuclear division* (GO:0007067), and *G1/S transition of mitotic cell cycle* (GO:0000082), suggesting that mRNAs involved in the cell cycle are more susceptible to PMI-related mRNA degradation in whole blood.

### Genotype may affect PMI related mRNA degradation in whole blood

Previous studies had reported that inter-individual variability exists in sensitivity to postmortem mRNA degradation of a given tissue^5, 11, 15^. However, little is known about the mechanism of this variability. In light of the potential effects of SNPs on the regulatory network and mRNA stability as well as postmortem mRNA degradation, we considered the possibility that an individual’s genotype may be a novel explanatory factor accounting for the inter-individual variability. We therefore further investigated the interaction between genotype and PMI to determine how genetic background influences on postmortem mRNA degradation in whole blood (see Methods).

**Figure 3:**
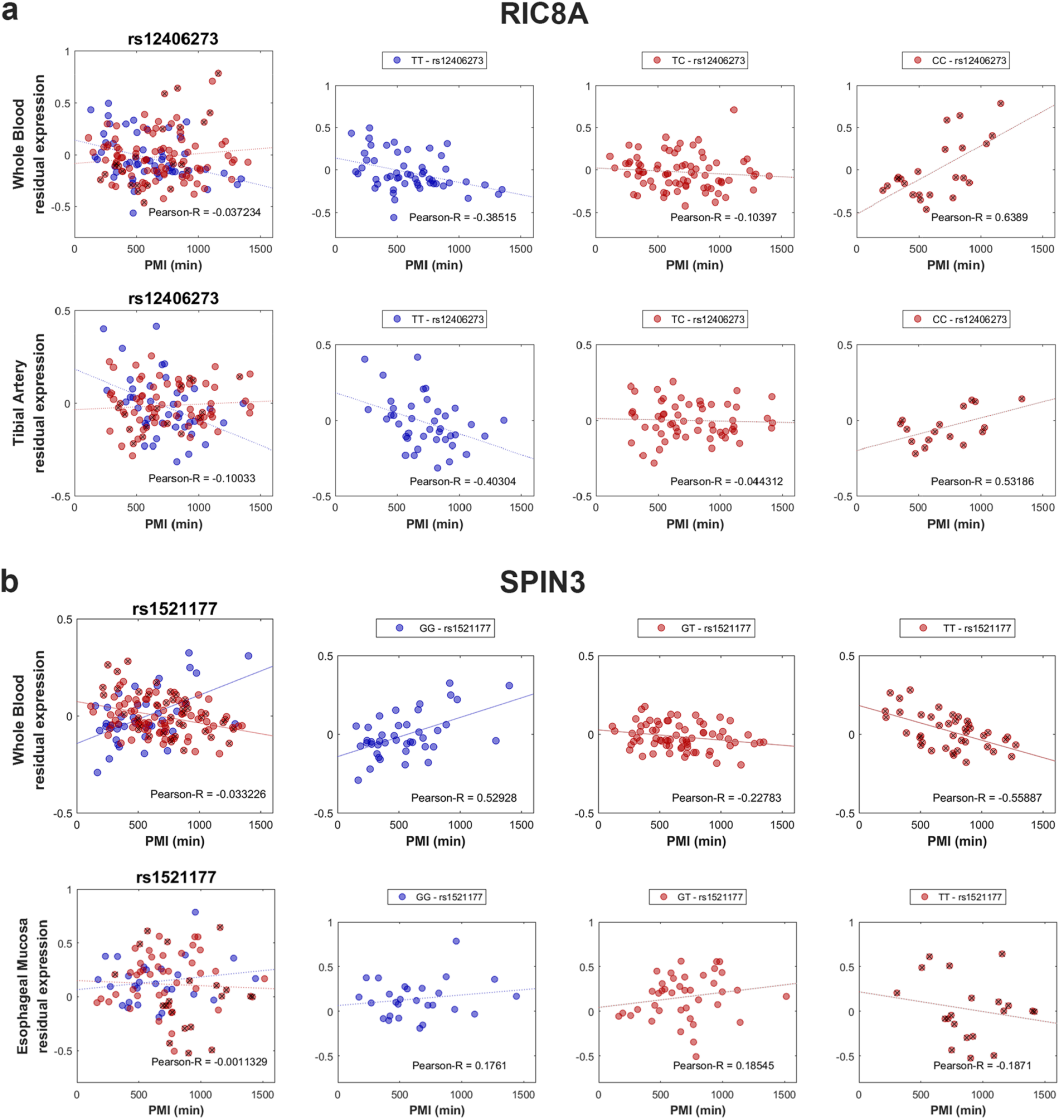
Examples of genotype-by-PMI interaction affecting the expression level of the gene. (**a**) The interaction between rs12406273 and PMI affecting RIC8A gene expression in tibial artery and whole blood. (**b**) The interaction of rs1521177 and PMI affecting SPIN3 gene expression in esophageal mucosa and whole blood. For each subplot, the larger panel on the left shows all samples, while the three smaller panels on the right show the samples with major allele homozygous, heterozygous and minor allele homozygous (with a cross) respectively.

In this case, we detected 740 significant (*P*-value < 4.75E-8) interactions. Among these interactions, 56% (418 of 740) were verified to a significant level in one or more additional tissue(s), indicating that genotype may affect the PMI related mRNA degradation. The comprehensive list of SNPs and genes are provided in Supplementary Data S3. Here, we show two typical examples. The genotypes of SNP rs12406273 can have different effects on expression of the RIC8 Guanine Nucleotide Exchange Factor A (RIC8A gene) in tibial artery and whole blood (Fig. 3a). For individuals with the TT genotype, there is a significant negative correlation of PMI and RIC8A expression. Individuals with TC genotypes show no correlation or correlation which is less strong, and individuals CC genotypes may even show a significant positive correlation. Another example of a relationship between the SNP genotype and the PMI-associated gene expression involves SPIN3 and the SNP rs1521177 in esophageal mucosa and whole blood (Fig. 3b). Once again, the GG genotype appears to confer a positive correlation with PMI, while the TT genotype is negatively correlated with PMI. Thus, we conclude that the same mRNA may undergo different rates of postmortem mRNA degradation for different individuals. These genotype-by-PMI interactions are probably linked with potential gene interactions whose mechanisms will require further study.

### Gene dispersion changed with prolonged PMI in human tissues

Considering the possibility that postmortem mRNA degradation may be a non-linear process, we examined the dispersion changes of gene expression in Short-PMI (S-PMI) and Long-PMI (L-PMI) groups in all tissues. Using Levene’s test, we identified 266 PMI-associated differentially variable (DV) genes which showed a significant difference in expression variance in S-PMI and L-PMI groups at FDR of 5% (Table 3). The distributions of identified PMI-associated DV genes in 15 human tissues were uneven in number, which is consistent with the distribution of previously identified PMI-associated genes, in that we identified more significant DV genes in the esophageal mucosa (74 genes) than in CNS tissues (0 genes). A comprehensive list of PMI-associated DV genes is provided in Supplementary Data S4.

**Table 3:** Numbers of PMI-associated differentially variable (DV) genes across 15 different human tissues.

Tissues	Decrease	Increase	Total
(1) Subcutaneous Adipose (*n* = 144)	8	2	**10**
(2) Aorta Artery (*n* = 85)	9	3	**12**
(3) Tibial Artery (*n* = 138)	0	1	**1**
(4) Cerebellum (*n* = 100)	0	0	**0**
(5) Cerebral Cortex (*n* = 83)	0	0	**0**
(6) Esophageal Mucosa (*n* = 101)	41	33	**74**
(7) Heart (*n* = 89)	23	1	**24**
(8) Lung (*n* = 125)	11	4	**15**
(9) Skeletal Muscle (*n* = 208)	15	8	**23**
(10) Tibial Nerve (*n* = 122)	1	0	**1**
(11) Pituitary (*n* = 81)	0	0	**0**
(12) Suprapubic Skin (*n* = 116)	0	0	**0**
(13) Lower leg Skin (*n* = 171)	3	0	**3**
(14) Thyroid (*n* = 116)	43	4	**47**
(15) Whole Blood (*n* = 183)	53	12	**65**

**Figure 4:**
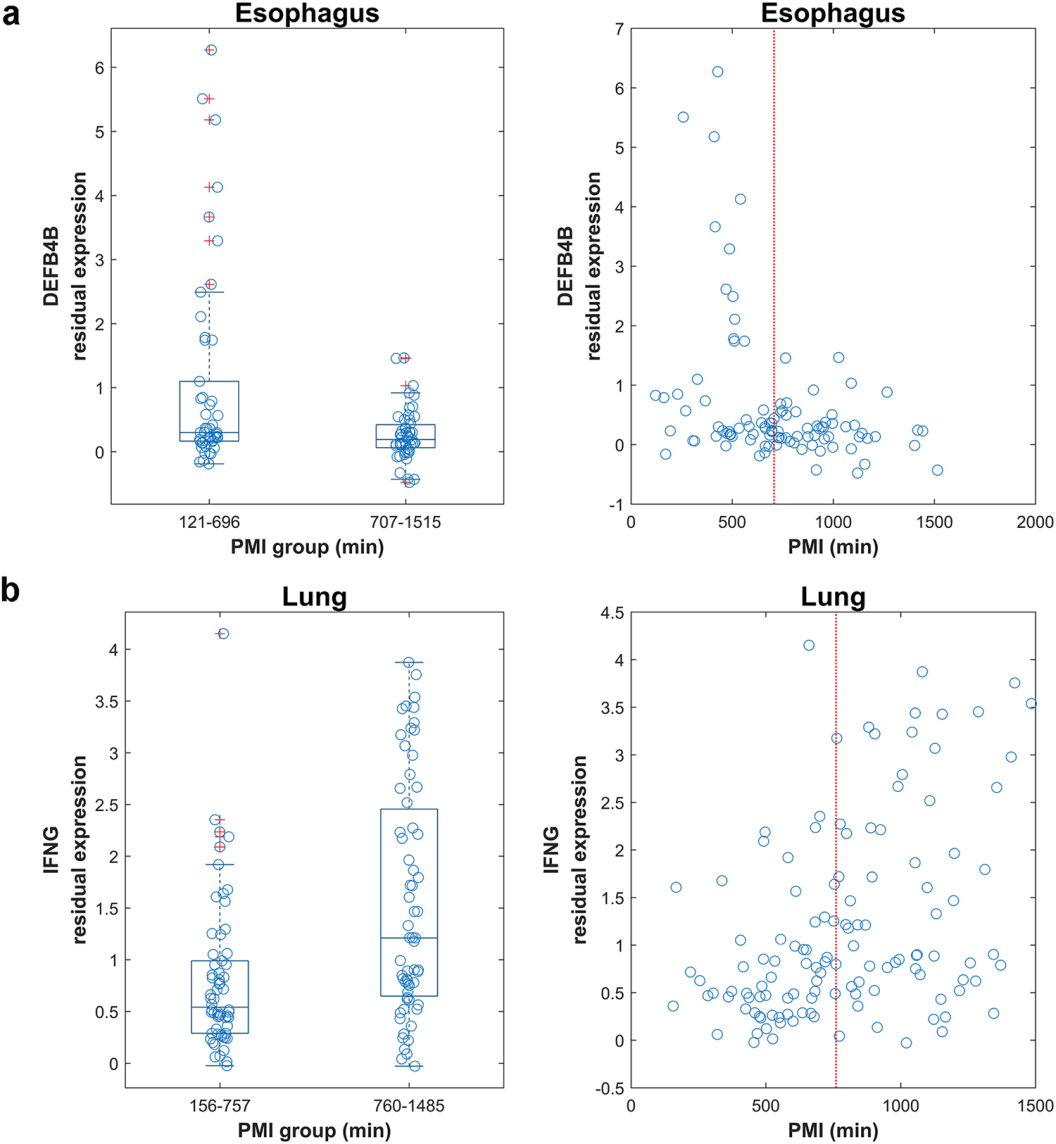
Examples of differential gene expression dispersion among PMI groups. (**a**) Increased gene expression variance of DEFB4B in esophageal mucosa between PMI groups (left:121–696 mins vs. 707–1,515 mins; right: all samples). (**b**) Decreased gene expression variance of IFNG in lung between PMI groups (left:156–757 mins vs. 760–1,485 mins; right: all samples). Each PMI group was plotted with jitter along the x-axis to show samples of different PMI. The red dotted line represents the median PMI of all samples.

Of these 266 PMI-associated DV genes, the expression variance of the majority (206 genes, 77%) decreased in the L-PMI group, while the others increased, supporting the common sense argument that postmortem mRNA degradation increases with prolonged PMI. For example, DEFB4B (Defensin Beta 4B) is an antibiotic peptide which plays a crucial role in host defense against microorganisms by permeabilizing bacterial membranes^24^. The expression dispersion of DEFB4B in esophageal mucosa is more pronounced in the S-PMI group than in the L-PMI group, and the residual expression tends to be zero with prolonged PMI (Fig. 4a). However, a few genes show an abnormal increase in expression variance with prolonged PMI. To take another example, IFNG (Interferon Gamma) is a cytokine that is critical for innate and adaptive immunity against viral, bacterial and protozoal infections. The expression dispersion of IFNG in the lung is more pronounced in the L-PMI than in the S-PMI group, and its residual expression tends to increase with prolonged PMI (Fig. 4b), implying the biological activity of IFNG would persist for a prolonged time (almost 24 hours) in lung tissues after death.

## Discussion

Despite the increasing demand for postmortem human tissue in many areas of research, collecting tissue samples without delay is challenging due to numerous factors, including legal issues, the expenditure of time and difficulty of collection, and ethical concerns. Delay in collecting and securing specimens, defined as the postmortem interval (PMI), brings an inevitable bias to gene expression research. To address this problem, we used the GTEx RNA-seq data set to investigate PMI-associated genes and provide a more comprehensive picture of mRNA degradation in diverse human postmortem tissues.

Our study identified 7,546 distinct PMI-associated genes with postmortem up- or down-regulation in 15 human tissues. We found that the sensitivity of different tissues to PMI-associated mRNA degradation shows distinct gene to gene differences, and in a given tissue, numbers of PMI-associated genes vary dramatically from none to 2,763. For example, mRNAs in the central nervous system show more stability than those in the digestive tract tissues (e.g., esophageal mucosa), which is consistent with previous studies^11, 25^. Of particular interest, we found that even different regions in the same tissue may have significantly different sensitivities to postmortem mRNA degradation, and differing numbers of PMI-associated genes may be identified. For instance, we identified 45 genes in suprapubic skin, but there were 636 genes in skin of the lower leg; and there were 194 genes in tibial artery contrasting with 1,413 in the aorta artery. Postmortem changes occur when the heart stops beating, resulting in a stagnation of blood flow in the veins or small blood vessels. It thus appears that small blood vessels are less susceptible to postmortem mRNA degradation than large blood vessels, which may explain the discrepancy in numbers of PMI-associated genes identified in the tibial artery and aorta artery. It is of note that the microcirculation continues working for some time after death, suggesting that tissues with abundant small blood vessels and microcirculation such as brain and lung tend to have little or no sensitivity to postmortem mRNA degradation. The discrepancy in numbers of PMI-associated genes in the lower leg skin and suprapubic skin can also be explained by the difference in the extent of microcirculation. These results are of particular importance in research utilizing mRNA from autopsies and may help determine the order in which organs are harvested.

In living cells, RNA degradation is a complex and highly regulated process^26^. For example, many cellular factors and mechanisms are involved in modulating the rate of mRNA degradation^27^, such as the deadenylation-dependent pathway, endonuclease-mediated mRNA decay, miRNA, and P-bodies^28^. In contrast, the mechanism for degradation of mRNA in most postmortem tissues is poorly understood. From our PMI-associated gene functional annotation analysis of eight tissues, we found that postmortem mRNA degradation is neither a random process nor a normal physiological condition. *Nuclear-transcribed mRNA catabolic process: nonsense-mediated decay* (GO:0000184), one of the known RNA degradation process, was identified in up-regulated enrichment annotations of three tissues, implying that postmortem mRNA degradation in these tissues may occur in this manner. In addition, we identified several tissue-specific GO terms and KEGG pathways in up- and down-regulated PMI-associated genes. This showed that mRNAs involved in protein synthesis are less susceptible to PMI-related RNA degradation than those involved in the immune response and in the proteasome. These findings are of particular importance for research focusing on specific gene expression using postmortem tissues. For example, postmortem blood should be collected earlier for analysis of gene expression involved in the cell cycle, as we have identified many cell cycle-related GO terms in down-regulated PMI-associated genes of whole blood.

We also investigated the interaction of genotype and PMI to explore the potential influence of genotype on PMI-related mRNA degradation. At a stringency *P*-value of 4.75E-8, we identified more than 700 PMI-genotype interactions in whole blood. These findings may provide direction for analysis of SNPs which potentially affect RNA stability or gene interaction, and hold promise for future studies. In addition, dispersion analysis using Levene’s test was performed in two PMI groups. The majority of DV genes we identified showed decreased expression variance with prolonged PMI, supporting the common sense concept that mRNA degradation increases in association with PMI. However, there are a few examples such as IFNG, which show an increase of expression variance in the L-PMI, indicating that some biological activities continue for some time after death.

Estimation of PMI is considered to be one of the most important tasks in the field of forensic medicine^29, 30^. We observed that the global pattern of gene expression can clearly be divided into two groups S-PMI and L-PMI. All of the 11 human tissues we evaluated showed significant *P*-values with the *t*-test. These results suggested that gene expression changes vary with PMI, and this characteristic might be used to estimate the PMI. Several studies have suggested that mRNA can be used to estimate PMI^3, 9, 19, 31, 32^. Our study thus provides novel direction for selection of the most suitable mRNA indicator for estimating PMI accurately. Specifically, we can use the most significantly correlated genes from the list of up-regulated genes to predict longer PMI, whereas using the list of most significantly decreased of the down-regulated genes predicts a shorter PMI.

To our knowledge, our analysis is currently the largest RNA-seq based transcriptome study of PMI affecting mRNA degradation in human postmortem tissues. In summary, we demonstrate that PMI-related mRNA degradation is tissue-specific, gene-specific, and genotype-dependent which thus allows a more comprehensive picture of PMI-associated gene expression across diverse human postmortem tissues to be drawn. These findings provide necessary information for both pathologists and molecular biologists to interpret gene expression patterns utilizing postmortem tissues.

## Methods

### GTEx Tissues and Expression Data

The GTEx data set (v6, October 2015 released) was downloaded from the GTEx project through dbGaP (https://dbgap.ncbi.nlm.nih.gov). In this data set, we selected 2,016 high-quality samples (RINs > 6.0) from 316 postmortem donors for study. For detailed information regarding sample collection, RNA sequencing, and the data processing pipeline refer to the GTEx Consortium paper^33^.

Subject-level variables, including age, gender, and body mass index (BMI) were obtained from the GTEx Portal (GTEx_Data_V6_Annotations_Subject Phenotypes DS.txt). Sample-level variable (SMTSISCH) was chosen to represent the postmortem interval and was obtained from the GTEx Portal (GTEx_Data_V6_Annotations_Sample Attributes DS.txt). Samples for which SMTSISCH was less than zero or was missing were excluded from further analysis. As we would have to detect the genotype-by-PMI interaction on gene expression, tissue samples from donors of non-European descent were filtered out.

We extracted the whole-gene level RPKM (Reads Per Kilobase of transcript per Million mapped reads) values for 18,763 protein-coding genes. The data for different tissues were quantile normalized, and log_2_-transformed, respectively. 20% of genes showing low expression were then excluded from data analysis based on their mean expression levels across samples in each tissue. Finally, 15,010 genes passed filtration. We confined our analysis to tissues with expression data for at least 80 samples, resulting in a total of 2,016 samples and 15 tissues. The sample size for each tissue ranged from 81 to 208 (Table 1).

### Correcting the confounding factors using the PEER algorithm

To correct the known covariates as well as infer hidden data structures in the GTEx expression data, we employed a two-step approach^34^ based on the PEER algorithm^35^ prior to regression analysis. PEER was first used to unearth patterns of common variation across the whole data set and create up to 15 assumed global hidden factors for each tissue. The known covariates, including the donor’s age, gender, and BMI together with the PMI for all samples from 15 tissues were contained in PEER models. It is of note that PMI was also included to enable PEER to discover correlated patterns across global structured data. Next, the correlation between each of the 15 constructed factors and PMI was tested with the data set of each tissue. Factors showing a Pearson’s correlation or Spearman’s rank correlation test *P*-value smaller than 0.05 were excluded. The remaining factors along with non-PMI covariates were used as a new set of covariates in the regression analysis. The residual values of the regression were used as corrected gene expression data in the further analysis.

### Multiple linear regression model for PMI-associated gene detection

For each tissue, we modeled gene expression using the following multiple linear regression model:

1 $${Y}_{ij}={\mu }_{j}+{\alpha }_{j}PM{I}_{i}+{\beta }_{j}Ag{e}_{i}+{\gamma }_{j}BM{I}_{i}+{\delta }_{j}Gende{r}_{i}+\sum _{k=1}^{N}{\theta }_{jk}P{C}_{ki}+{\varepsilon }_{ij}$$

where *Y*_*ij*_ is the expression level of gene *j* in the sample *i*; *PMI*_*i*_ which denotes the postmortem interval for sample *i* with regression coefficient *α*_*j*_ for gene *j*; *Age*_*i*_ denotes the age of sample *i* with regression coefficient *β*_*j*_ for gene *j*; *BMI*_*i*_ denotes the BMI of sample *i* with regression coefficient *γ*_*j*_ for each gene *j*; *Gender*_*i*_ denotes the gender of sample *i* with regression coefficient *δ*_*j*_ for gene *j*; *PC*_*ki*_ (1 ≤ *k* ≤ *N*) denotes the value of the *k*-th hidden factors of the gene expression profile for the *i*-th sample with regression coefficient *θ*_*jk*_; *N* is the total number of factors uncorrelated with PMI; *ε*_*ij*_ is the error term, and *μ*_*j*_ is the regression intercept (for gene *j*).

We fitted the model with the fitlm function in the Statistics toolbox of MATLAB. For each gene, a least square approach was used to estimate the regression coefficients. If *α*_*j*_ was significantly deviated from 0, the gene *j* was considered to be PMI-associated. A gene was considered to be up-regulated (that is, the gene was degraded significantly more slowly than the mean degradation rate or even increasingly expressed) with PMI if *α* > 0 and down-regulated (that is, the gene was degraded significantly more quickly than the mean degradation rate) if *α* < 0.

Throughout this study, GO term enrichment analyses were carried out by using the functional annotation tool of DAVID Bioinformatics Resource Server (version 6.8)^36^. The false discovery rate (FDR) adjustment for *P*-values was made using the Benjamin-Hochberg procedure^37^. An FDR less than 0.05 was considered as the threshold for significance unless otherwise specified.

### Effect of sample size, bootstrapping and permutation analysis

To evaluate the number of false positives that could be involved in our PMI-associated genes at FDR of 5%, we adopted an approach similar to that of Yang and Huang^38^. For each gene, we randomly permuted the sample PMI and repeated the identification procedure 10,000 times. We counted the number of occasions that the *P*-value was less than the original one, and included the genes whose number was smaller than 500 in our final results (Table 1).

To examine the effect of sample size, for each tissue we randomly selected samples of sizes ranging from 20 to the maximum number with ten additional samples added each time (Fig. S2) and then bootstrapped 100 times. As expected, larger sample size increases the power of identifying PMI-associated genes (e.g., the number of PMI-associated gene increases more than 100-fold in whole blood when sample size increases from 40 to 180). To correct the effect of sample size, we randomly selected the samples of size 80 (that is, the minimal sample size in 15 tissues) and bootstrapped 100 times, ensuring that our PMI-associated genes were not sensitive to a particular sample size. We counted the average number of PMI-associated genes in 100 repetitions of bootstrapping (Table S2).

### Genotype-by-PMI interaction on PMI related mRNA degradation

The genotype data for the 450 donors was obtained from dbGaP (http://www.ncbi.nlm.nih.gov/gap). For each polymorphic site, an individual’s genotype was denoted with 0, 1 or 2 based on the number of alternative alleles. SNPs with minor allele frequency (MAF) less than 15% were filtered out, resulting in a total of 1,051,649 SNPs retained for further testing.

2 $$\begin{array}{rcl}{Y}_{ij} & = & {\mu }_{j}+{\alpha }_{j}PM{I}_{i}+SN{P}_{s}+{\beta }_{j}Ag{e}_{i}+{\gamma }_{j}BM{I}_{i}\\  &  & +\,{\delta }_{j}Gende{r}_{i}+\sum _{k=1}^{N}{\theta }_{jk}P{C}_{ki}+PM{I}_{i}\cdot SN{P}_{s}+{\varepsilon }_{ij}\end{array}$$

To discover the Genotype-by-PMI interaction on PMI related mRNA degradation, we added the genotype (*SNP*_*S*_, 1 ≤ *s* ≤ 1,051,649) and genotype-by-PMI interaction terms (*PMI*_*i*_·*SNP*_*s*_) to the linear regression model described above. As a factor contributing to the gene expression variance, the significance of this interaction term was assessed for each gene after the model was fitted. To insure the overall computing time would be feasible, we randomly selected 2,000 genes in whole blood. All the computations were performed at the high-performance computing cluster of Peking University Institute of Systems Biomedicine.

To validate our results, we re-examined the significant interactions identified in whole blood using the other tissues from the GTEx dataset and permuted the PMI 10,000 times. We counted the number of occasions that the *P*-value was less than the original one, and only included the interactions whose number was smaller than 500.

### Test for expression heteroscedasticity between PMI groups

To compare the variance homogeneity of gene expression in Short-PMI (S-PMI) and Long-PMI (L-PMI) groups, we performed the Levene’s tests^39^ to determine whether the gene expression levels of different PMI groups have similar deviations from the group means. Let *x*_*kj*_ be a set of *j* = 1, …, *n*_*k*_ observations in each of *k* = 1, …, *g* PMI groups. The Levene’s test statistic is the ANOVA *F*-ratio comparing the *g* groups, calculated on the absolute deviations ${z}_{kj}=|{x}_{kj}-{\bar{x}}_{k}|$, where ${\bar{x}}_{k}=\frac{1}{{n}_{k}}\sum _{j=1}^{{n}_{k}}{x}_{kj}$ is the group means. The output value is the probability that at least one of the samples in the test has a significantly different variance, for which values less than 0.05 were considered statistically significant.

### Statistical analysis and data availability

Statistical computing was performed using MATLAB (v2016b) and R (v3.3, https://www.r-project.org/). The GTEx genotype and RNA sequencing data were downloaded from dbGaP (http://www.ncbi.nlm.nih.gov/gap), with study Accession number no.phs000424.v6.p1.

## Electronic supplementary material

Supplementary Information

Supplementary Dataset 1

Supplementary Dataset 2

Supplementary Dataset 3

Supplementary Dataset 4
