# Supplementary material for: Systematic analysis of gene expression patterns associated with postmortem interval in human tissues
Source: Sci Rep. 2017 Jul 14;7:5435. doi: 10.1038/s41598-017-05882-0 (PMC5511187; doi:10.1038/s41598-017-05882-0)
Supplement: Supplementary file 1 — Supplementary Information [file 41598_2017_5882_MOESM1_ESM.pdf]

List of Supplementary Files submit to *Scientific Reports*

Systematic analysis of gene expression patterns associated with postmortem interval in human tissues

Yizhang Zhu[1,2], Likun Wang[1], Yuxin Yin[1,2*], Ence Yang[1,3*]

[1]Institute of Systems Biomedicine, School of Basic Medical Sciences, Peking University Health Science Center, Beijing, 100191, China

[2]Department of Pathology, School of Basic Medical Sciences, Peking University Health Science Center, Beijing, 100191, China

[3]Department of Microbiology, School of Basic Medical Sciences, Peking University Health Science Center, Beijing, 100191, China

*Author for correspondence
Email: yangence@bjmu.edu.cn or yinyuxin@hsc.pku.edu.cn

Supplementary Figures

(a)

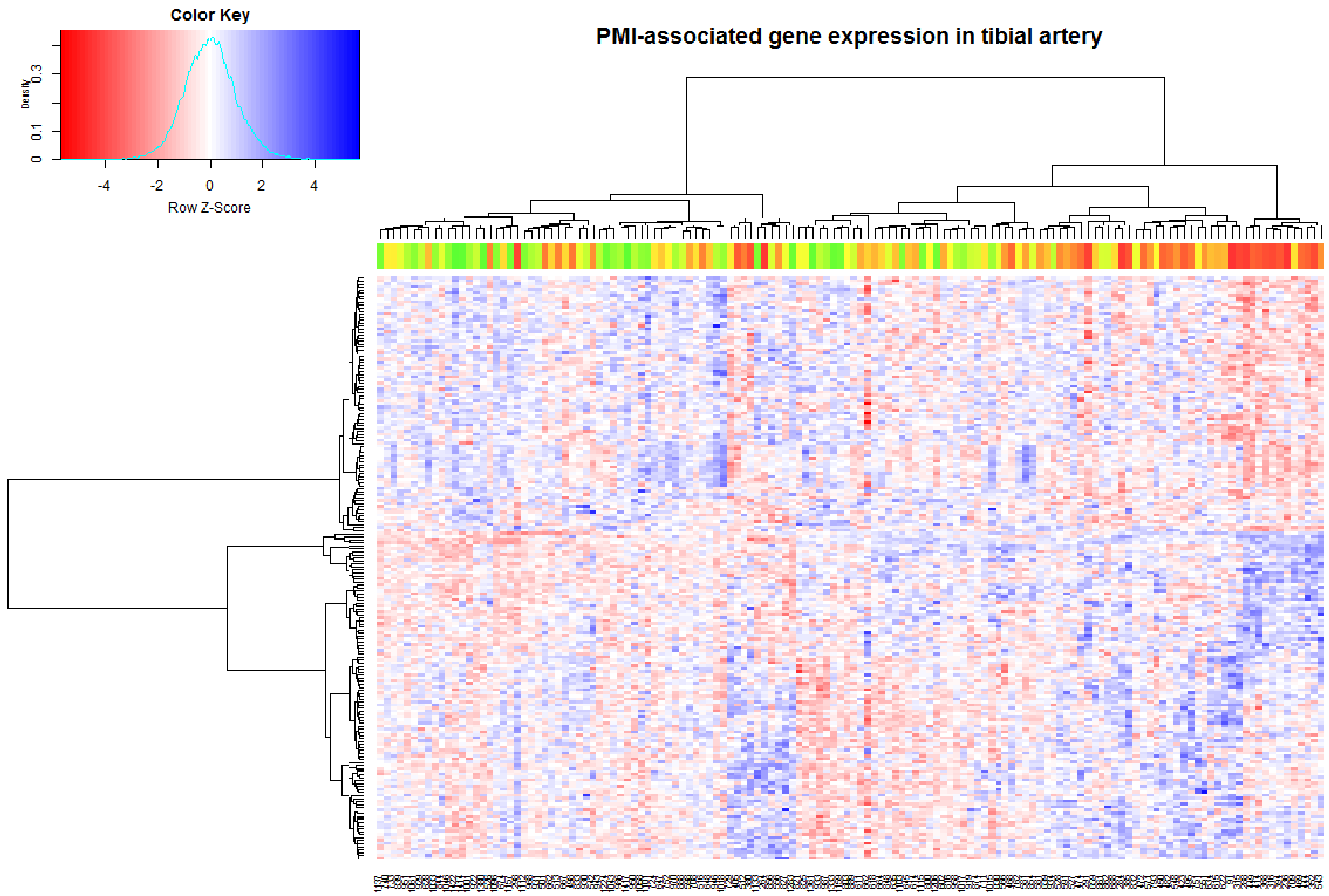

(b)

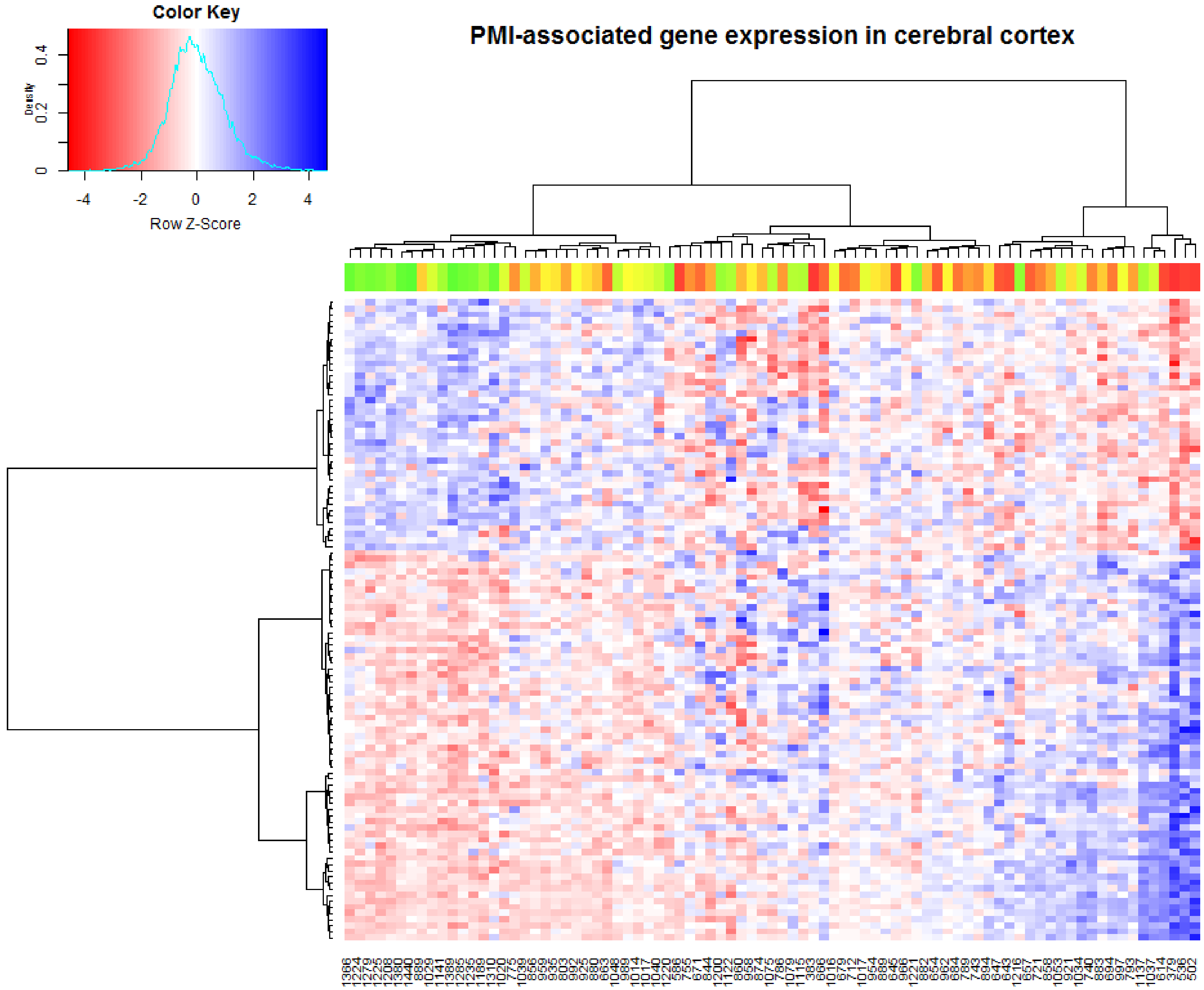
Color Key
Density
0
0.2
0.4
-4
-2
0
2
4
Row Z-Score
PMI-associated gene expression in cerebral cortex

(c)

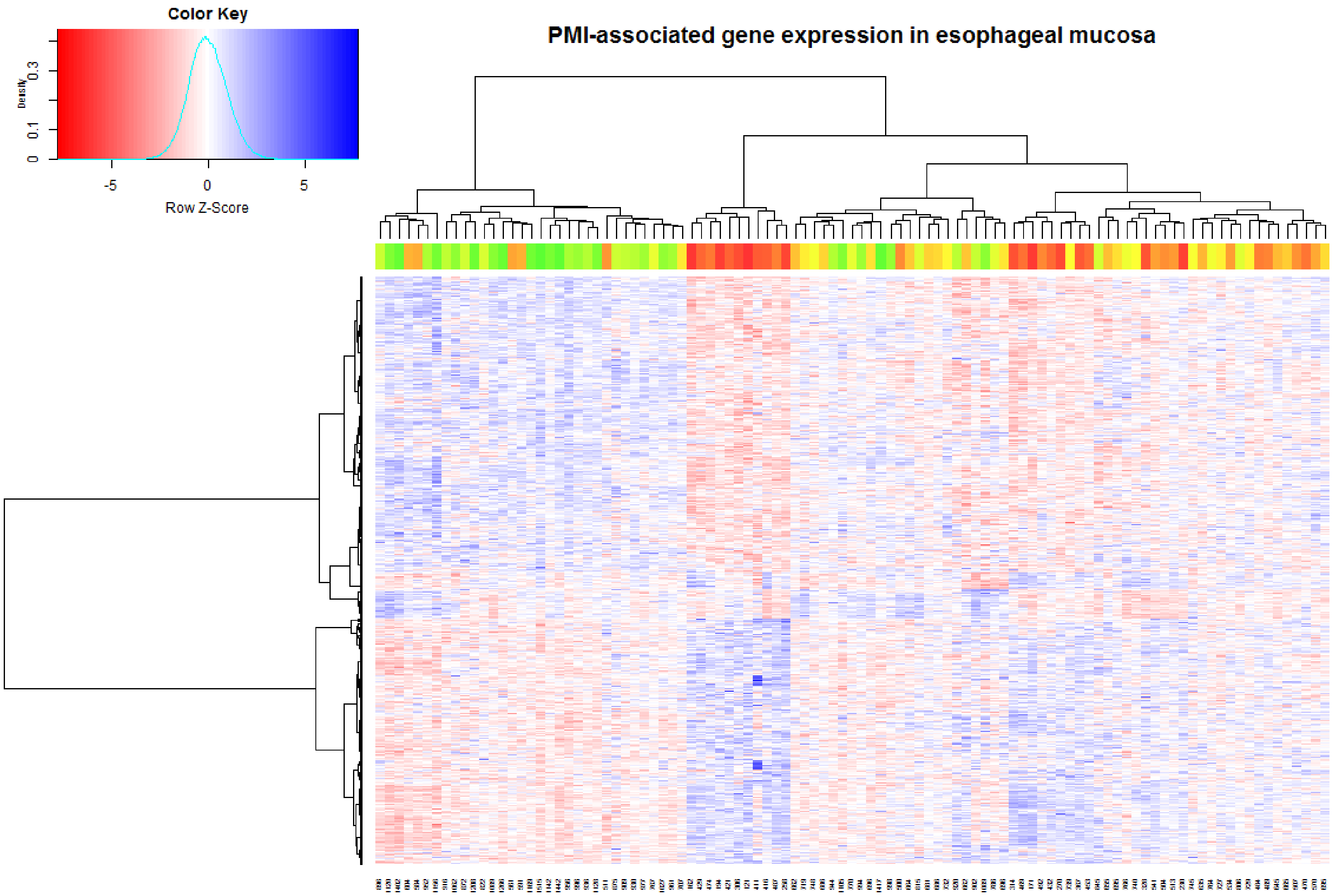

Color Key
Density
0
0.1
0.3
-5
0
5
Row Z-Score
PMI-associated gene expression in esophageal mucosa

(d)

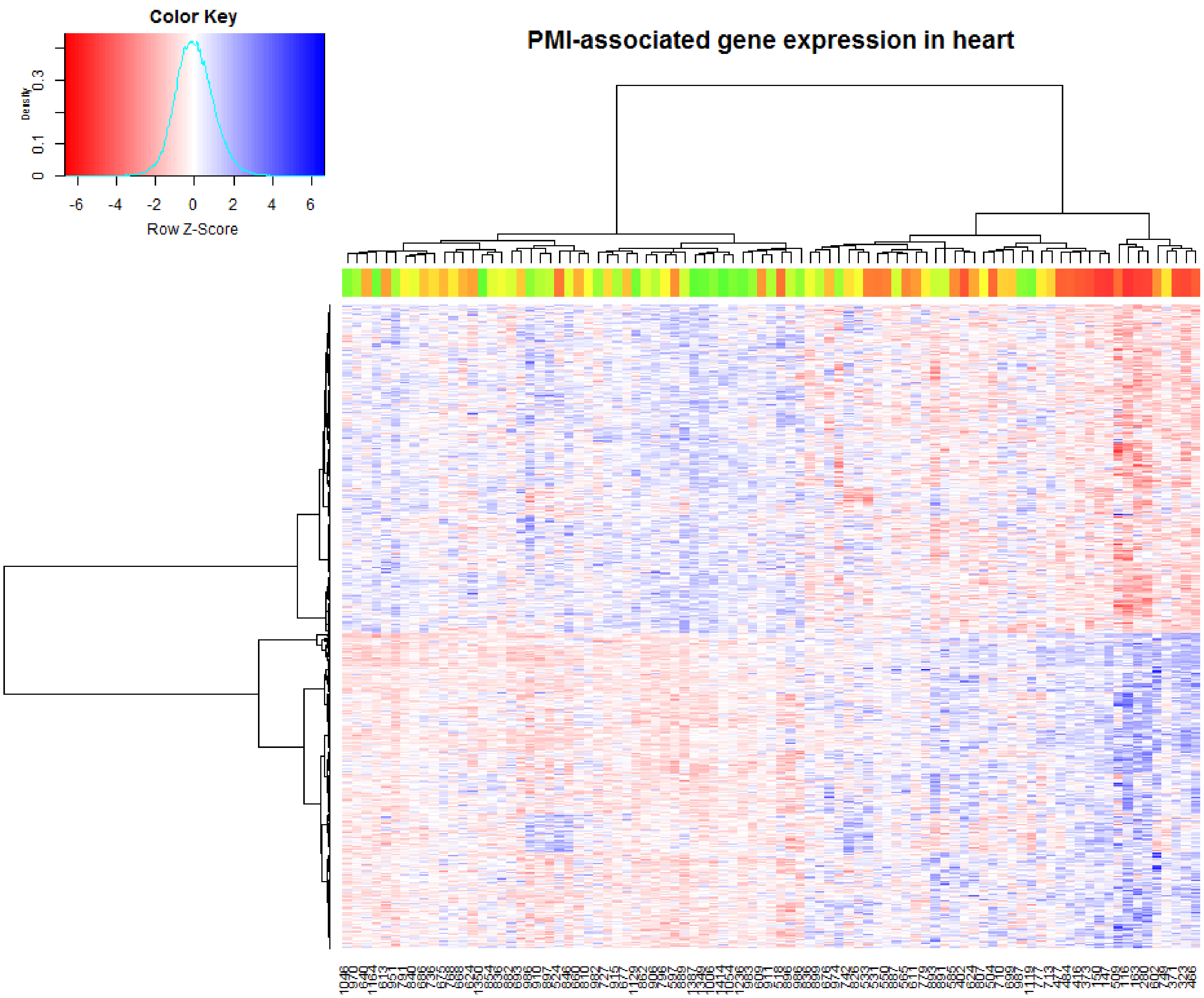
Color Key
Density
0
0.1
0.3
-6
-4
-2
0
2
4
6
Row Z-Score
PMI-associated gene expression in heart

(e)

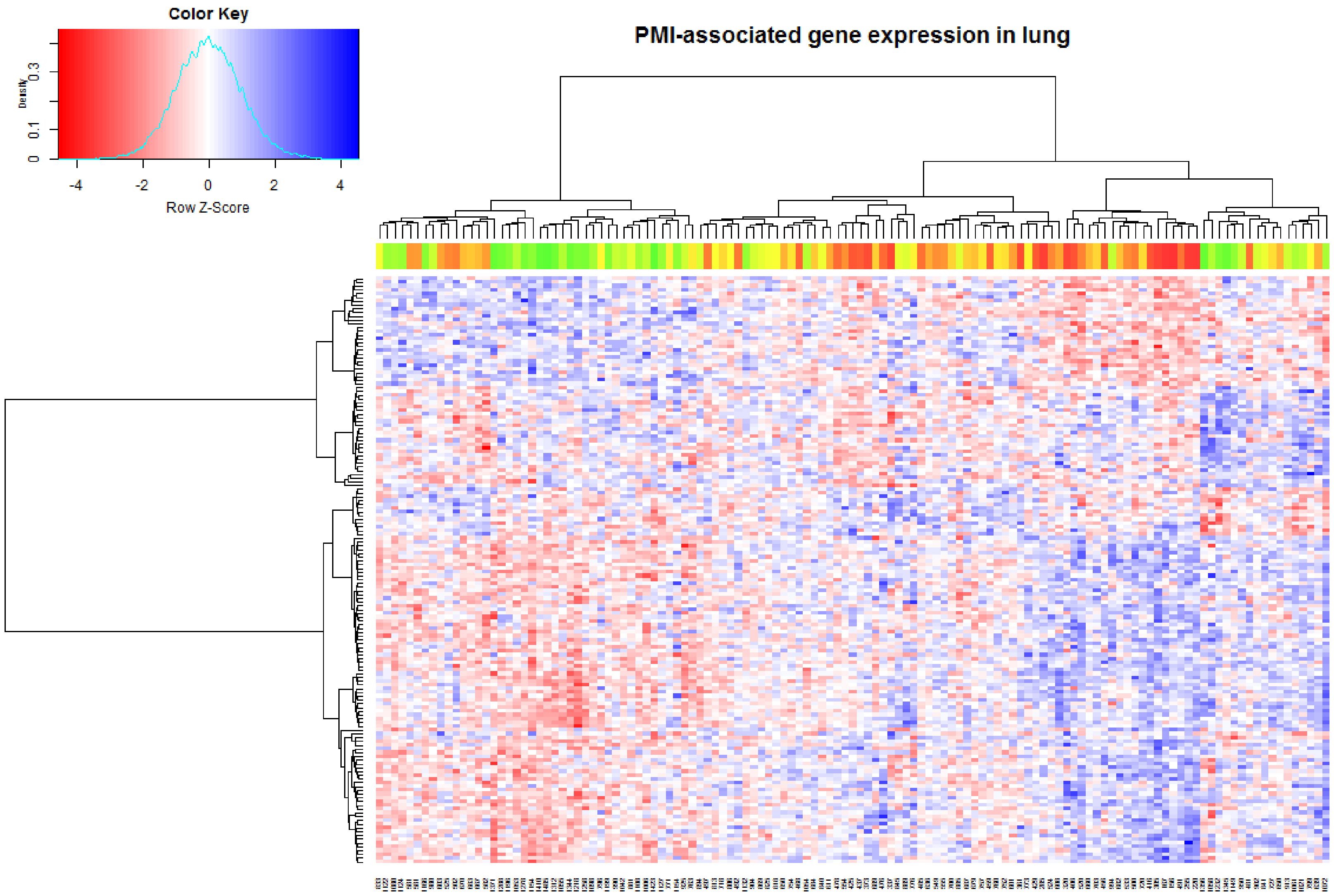
Color Key
Density
Row Z-Score
-4
-2
0
2
4
0
0.1
0.3
PMI-associated gene expression in lung

(f)

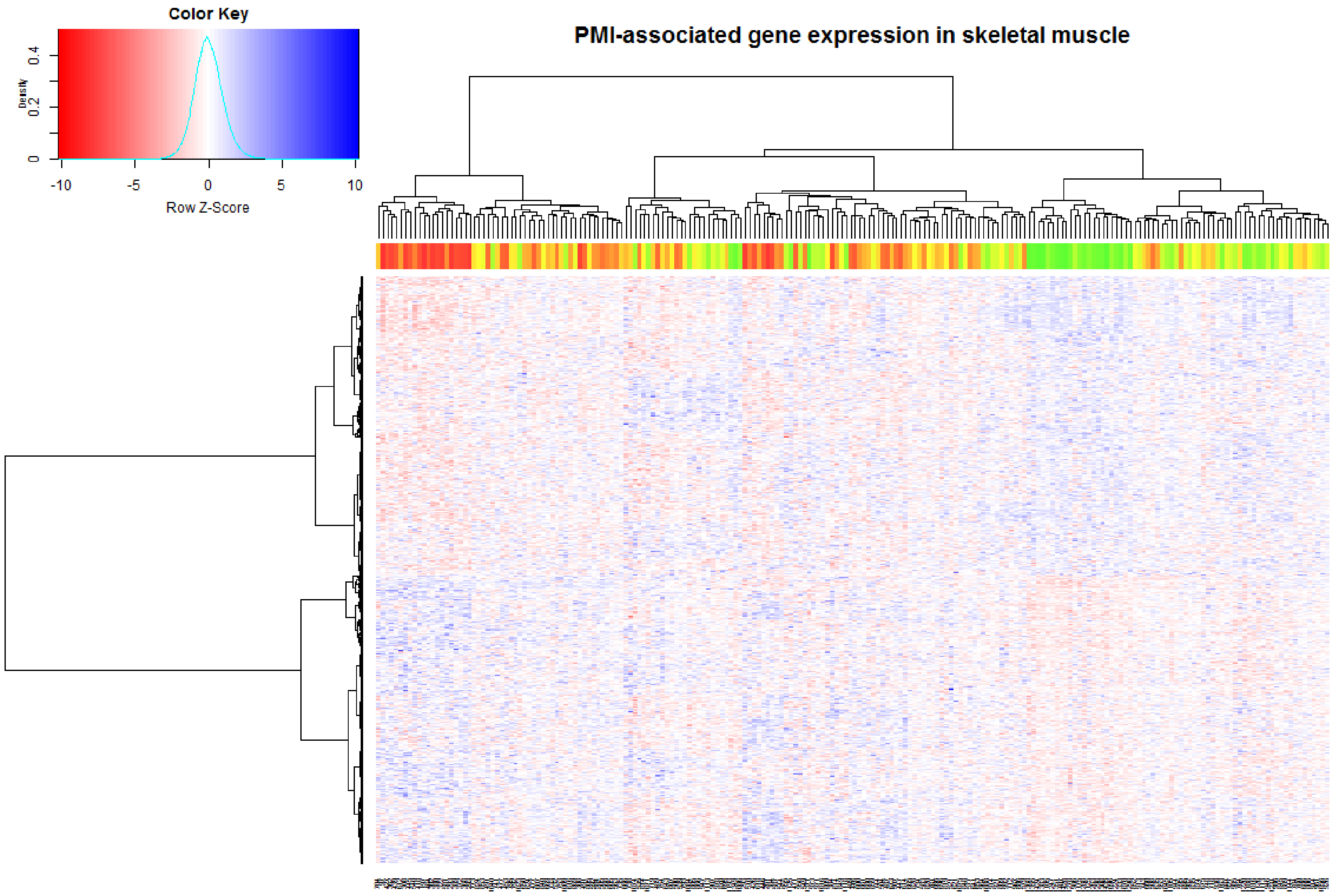
Color Key
Density
0
0.2
0.4
-10
-5
0
5
10
Row Z-Score
PMI-associated gene expression in skeletal muscle

(g)

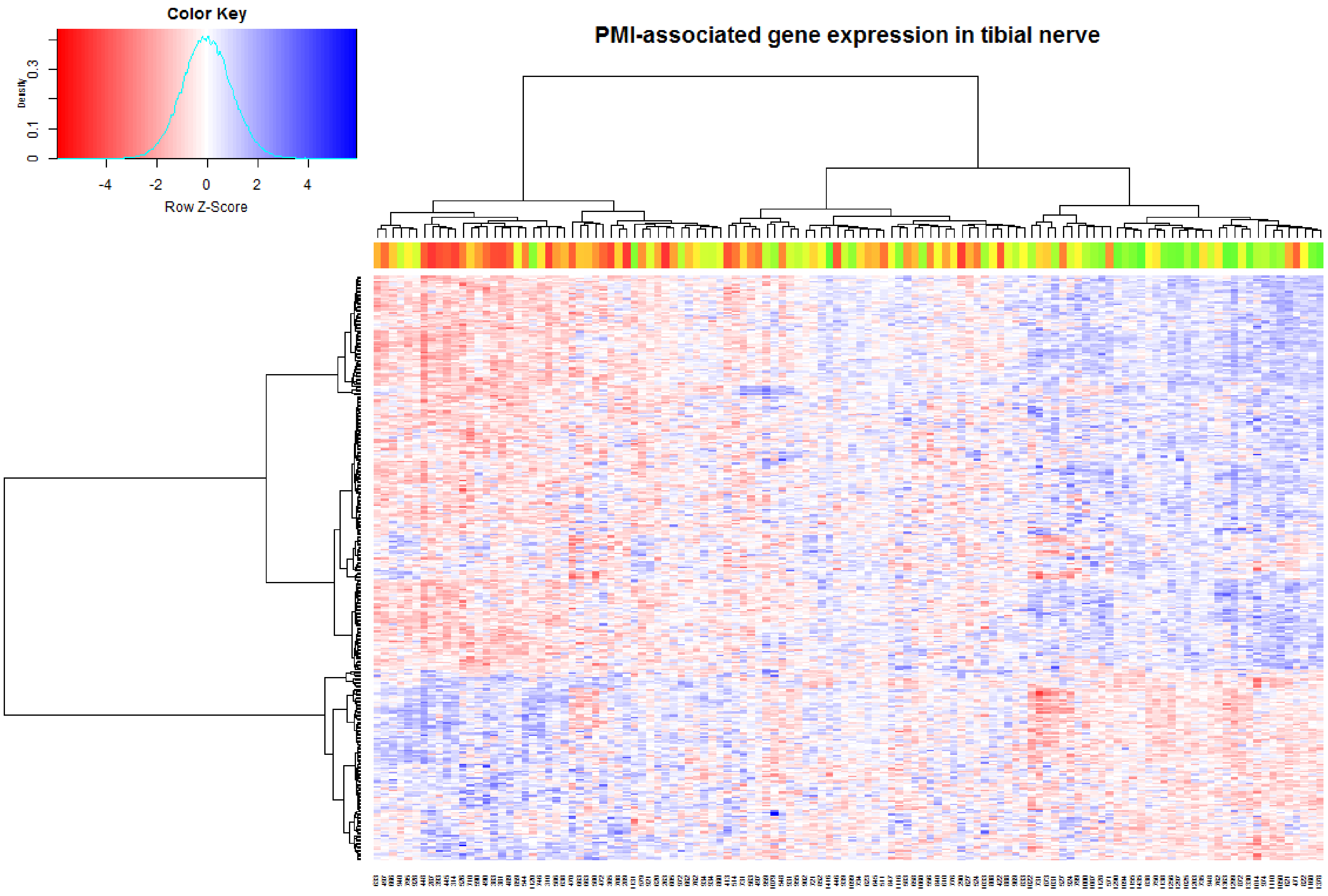
Color Key
Density
0 0.1 0.3
-4 -2 0 2 4
Row Z-Score
PMI-associated gene expression in tibial nerve

(h)

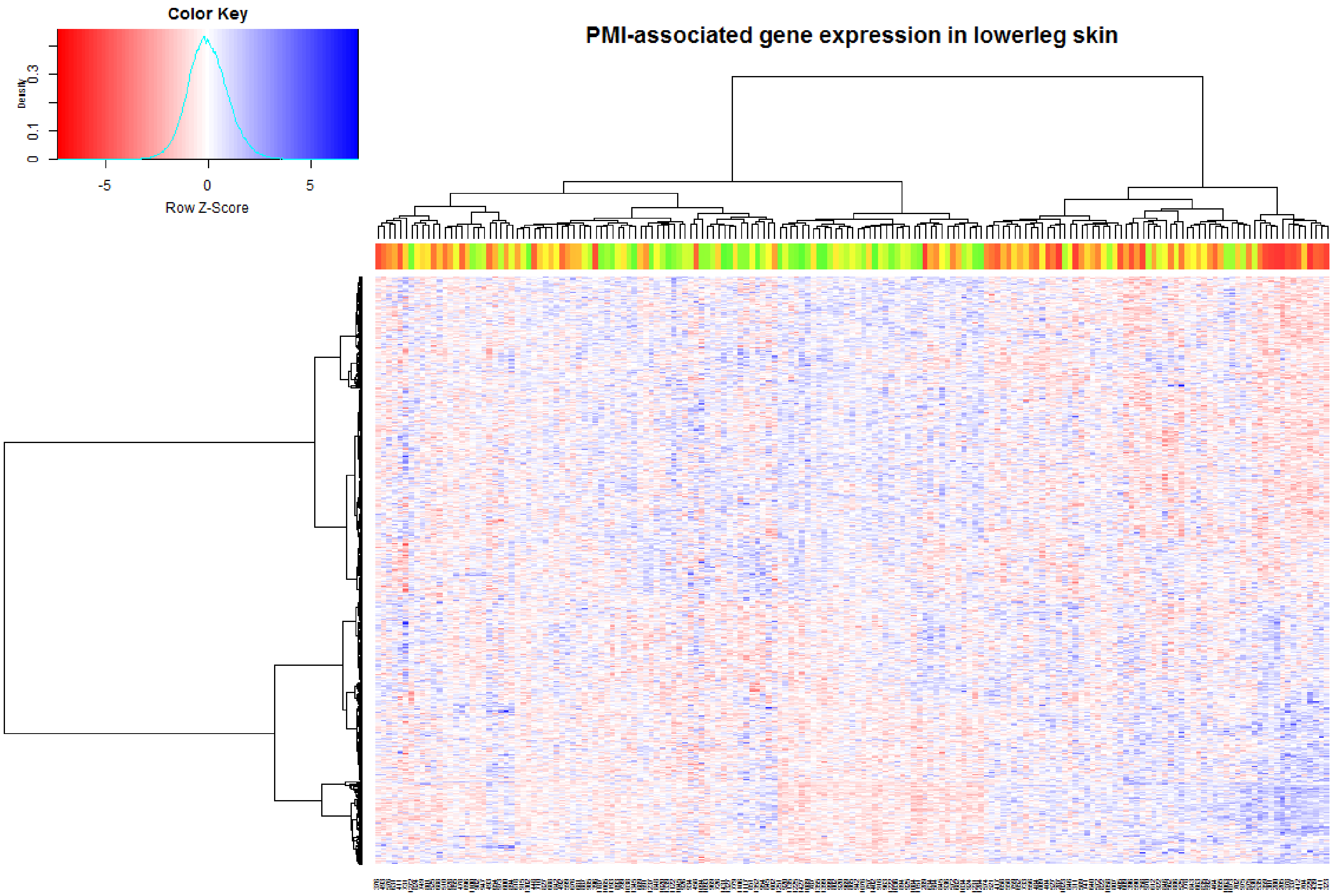

Color Key
Density
0
0.1
0.3
-5
0
5
Row Z-Score
PMI-associated gene expression in lowerleg skin

(i)

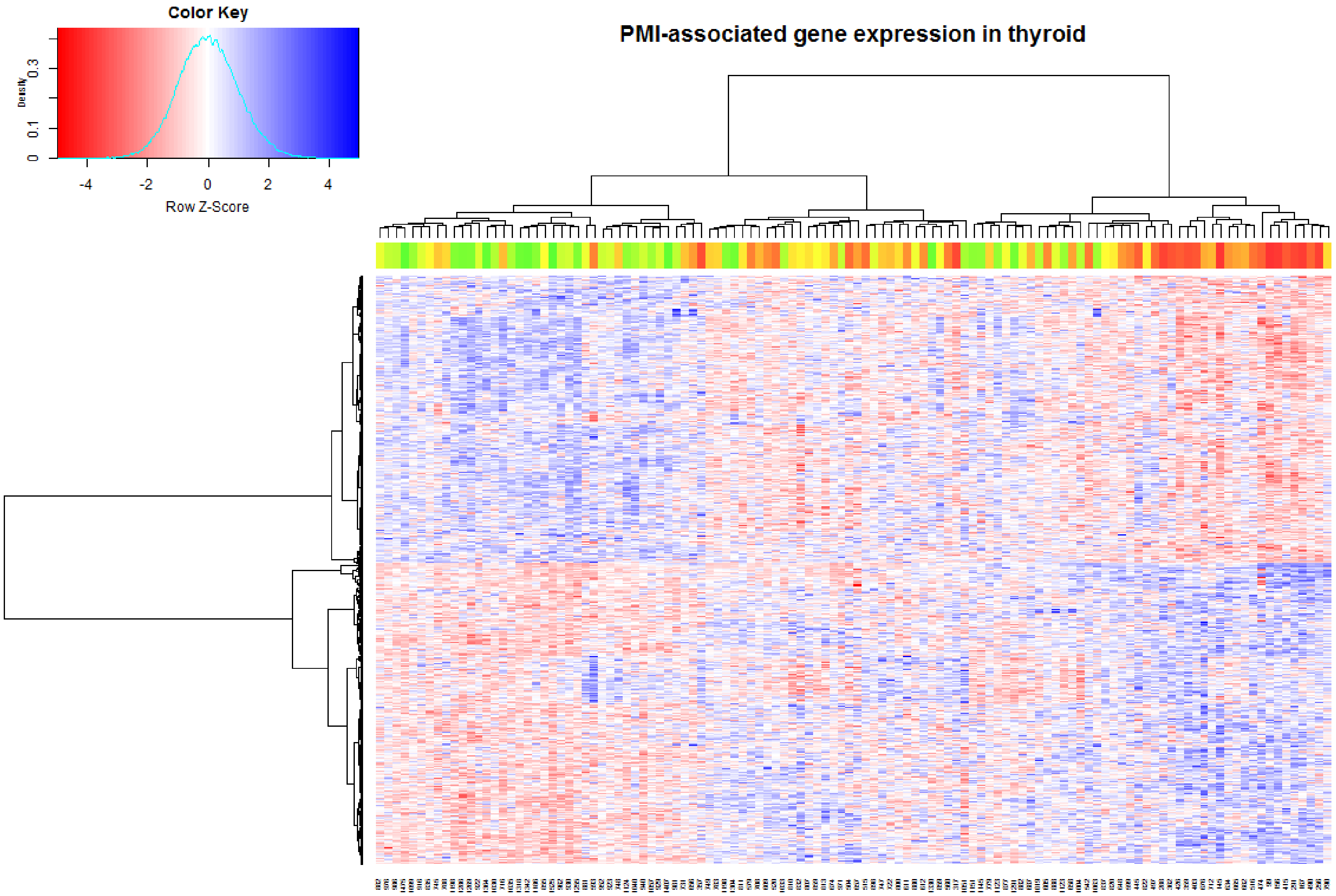

Color Key
Density
0
0.1
0.3
-4
-2
0
2
4
Row Z-Score
PMI-associated gene expression in thyroid

(j)

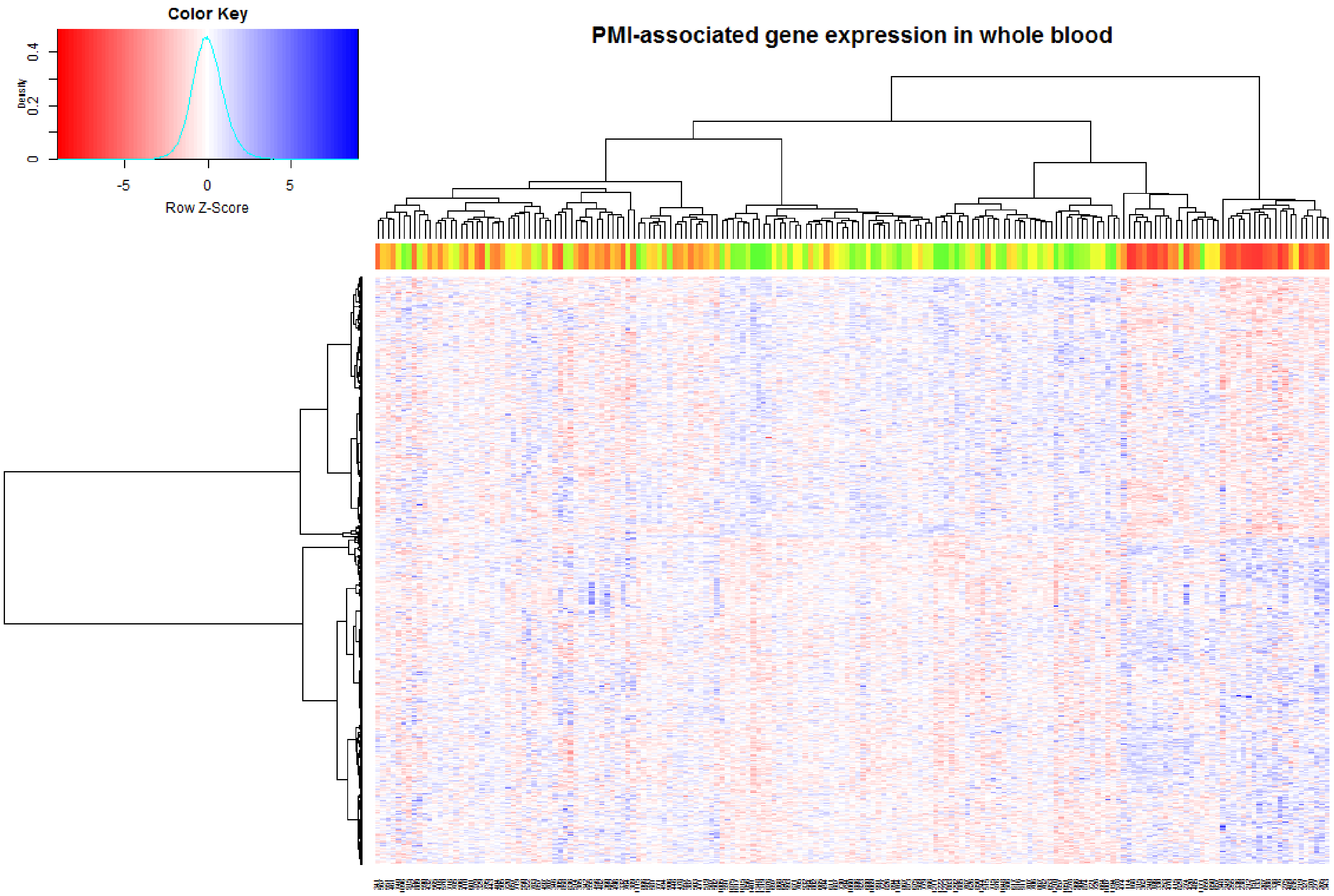

Fig. S1. PMI-associated gene expression in (a) Tibial Artery, (b) Cerebral Cortex, (c) Esophageal Mucosa, (d) Heart, (e) Lung, (f) Skeletal Muscle, (g) Nerve, (h) Lower leg Skin, (i) Thyroid, (j) Whole Blood.

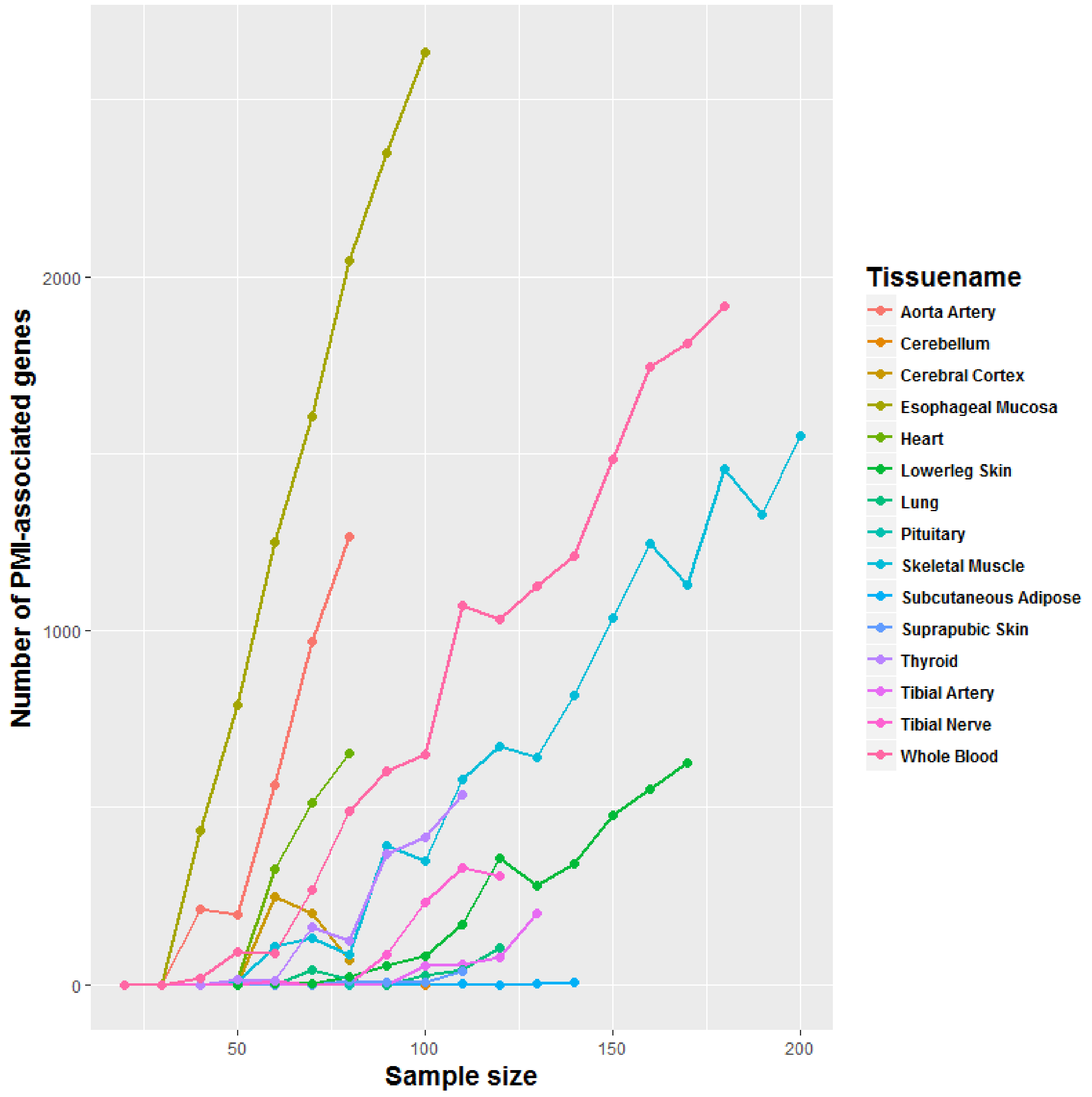

Fig. S2. Effect of sample size on inferring PMI-associated genes in 15 human tissues. The x-axis indicates the sample size and y-axis indicates the average number of PMI-associated genes in 100 repetitions of bootstrapping.

Supplementary Tables

Tissues	S-PMI		L-PMI		*P*-value
	Mean PMI	Sample size	Mean PMI	Sample size	
**(1) Aorta Artery**	9.09	48	13.80	37	**6.16E-07**
**(2) Tibial Artery**	11.16	77	14.23	61	**1.22E-04**
**(3) Cerebral Cortex**	13.39	20	16.26	63	**5.89E-03**
**(4) Esophageal Mucosa**	10.18	68	16.19	33	**4.03E-09**
**(5) Heart**	10.06	41	14.67	48	**4.35E-07**
**(6) Lung**	11.30	83	16.83	42	**8.14E-09**
**(7) Skeletal Muscle**	9.48	54	14.56	154	**1.28E-13**
**(8) Tibial Nerve**	10.43	45	14.59	77	**3.88E-07**
**(9) Lower leg Skin**	10.24	62	14.90	109	**3.20E-11**
**(10) Thyroid**	10.80	44	15.12	72	**1.21E-05**
**(11) Whole Blood**	4.83	21	11.73	162	**2.20E-16**

Table S1. The difference of S-PMI and L-PMI expression groups in samples of eleven tissues by the Student's *t*-test.

Tissues	FDR<1%			FDR<5%			FDR<10%		
	up	down	total	up	down	total	up	down	total
**(1) Cerebellum (*n*=80)**	0	0	**0**	0	0	**0**	0	0	**0**
**(2) Pituitary (*n*=80)**	0	0	**0**	0	0	**0**	0	0	**0**
**(3) Subcutaneous Adipose (*n*=80)**	1	1	**1**	1	1	**2**	1	1	**3**
**(4) Suprapubic Skin (*n*=80)**	1	1	**2**	3	2	**5**	4	3	**6**
**(5) Lung (*n*=80)**	6	13	**18**	8	16	**25**	10	19	**28**
**(6) Tibial Artery (*n*=80)**	7	13	**20**	13	20	**32**	16	25	**42**
**(7) Cerebral Cortex (*n*=80)**	2	8	**10**	40	60	**100**	121	131	**251**
**(8) Tibial Nerve (*n*=80)**	53	22	**75**	70	36	**106**	77	44	**121**
**(9) Lower leg Skin (*n*=80)**	37	33	**70**	69	55	**124**	95	72	**167**
**(10) Thyroid (*n*=80)**	89	85	**174**	122	122	**244**	144	142	**286**
**(11) Skeletal Muscle (*n*=80)**	144	115	**259**	184	152	**336**	211	173	**384**
**(12) Heart (*n*=80)**	175	180	**355**	374	366	**740**	515	516	**1031**
**(13) Whole Blood (*n*=80)**	262	306	**568**	340	407	**748**	388	457	**846**
**(14) Aorta Artery (*n*=80)**	400	468	**868**	639	675	**1314**	779	787	**1565**
**(15) Esophageal Mucosa (*n*=80)**	967	826	**1793**	1289	986	**2276**	1458	1073	**2531**

Table S2. Identification of PMI-associated genes by randomly subsampling samples to 80 (the minimal sample size) in 15 human tissues. Columns "up" and "down" list the number of positive correlation and negative correlation PMI-associated genes, respectively. Results derived from using three different FDR cutoffs (1%, 5%, and 10%) are shown.

Supplementary Data Legends

Data S1. The detail list of PMI-associated genes in 15 human tissues.

Data S2. The complete list of functional annotations of PMI-associated genes in eight tissues.

Data S3. The comprehensive list of Genotype-by-PMI interactions for 2,000 genes in whole blood.

Data S4. The complete list of PMI-associated DV genes.
